# Systematic Review and Meta‐Analysis of Audiometric Parameters for Assistive Hearing Technology for Adults and Children With Tympanic Membrane Perforation

**DOI:** 10.1111/coa.14295

**Published:** 2025-03-02

**Authors:** Thomas Hampton, Alan Sanderson, Kevin Mortimer, Mahmood Bhutta

**Affiliations:** ^1^ Department of Clinical Sciences Liverpool School of Tropical Medicine Liverpool UK; ^2^ Institute of Life Course and Medical Sciences University of Liverpool Liverpool UK; ^3^ Brighton & Sussex Medical School Brighton UK; ^4^ University Hospitals Sussex NHS Foundation Trust Brighton UK; ^5^ Cambridge Africa University of Cambridge Cambridge UK; ^6^ Department of Paediatrics and Child Health, School of Clinical Medicine, College of Health Sciences University of KwaZulu Natal Durban South Africa; ^7^ Department of Respiratory Medicine Liverpool University Hospitals NHS Foundation Trust Liverpool UK

## Abstract

**Introduction:**

Tympanic membrane perforation can cause hearing impairment with detrimental effects on communication and quality of life, and is a problem affecting an estimated 250 million people. To date, there is little analysis to inform public policy on options for assistive hearing technology rehabilitation in this group.

**Methods:**

We undertook a systematic review and meta‐analysis of six electronic databases registered with PROSPERO and reported in accordance with PRISMA 2020 standards. The primary outcome was the mean air and bone conduction hearing threshold associated with tympanic perforation.

**Results:**

Of 720 studies identified, 16 contained data for meta‐analysis. Mean air conduction threshold was 48.3 dB HL in adults and 31.9 dB HL in children. Mean bone conduction was 26.6 dB HL in adults and 9.5 dB HL in children. The prediction interval was −1.7 to 46.7 dB HL for bone conduction and 15.0–70.5 dB HL for air conduction.

**Conclusions:**

The majority of adults and children with tympanic perforation have air conduction thresholds within the range of rehabilitation with air conduction hearing aids. The majority also have good sensorineural hearing reserve, meaning bone conduction devices are also suitable. Our analysis can guide the development of affordable technology for the rehabilitation of those with tympanic perforation.

**Summary**

- *Question*: What hearing threshold level would you expect for adults and children with tympanic membrane perforation, and what type of assistive technology could help them?
- *Findings*: In this systematic review and meta‐analysis that assessed 720 studies, adults with a tympanic perforation had a mean air conduction threshold of 48.3 dB HL and children of 31.9 dB HL.
- *Additional findings*: Mean bone conduction thresholds in adults were 26.6 dB HL and in children 9.5 dB HL.
- *Meaning*: Most children and adults could benefit from air conduction devices, and most children and adults up to age 58 years could also benefit from bone conduction devices.
- *Future*: These findings can guide the provision and further development of affordable assistive hearing technology for adults and children with tympanic membrane perforation.

## 1 Introduction

Tympanic membrane (TM) or ear drum perforation is a condition with reported prevalence from 0.45% to 3% across the world [1, 2]. Such perforations may be associated with hearing loss or with recurrent or persistent ear discharge, with the latter termed chronic suppurative otitis media (CSOM) [3]. CSOM has been modelled to have a global prevalence of 250 million people and disproportionately affects socioeconomically disadvantaged populations, with those in high‐income settings typically having a prevalence of one in 200, but countries with lower socio‐economic status reportedly having as much as 25 times higher [4].

The TM has an integral role in transforming sound pressure from acoustic energy in the air into ossicular vibration [5], hence tympanic perforation causes conductive hearing loss. Human cadaveric studies report that if the whole TM is missing, this is associated with 27 dB hearing loss [6], but real‐world studies report variable severity. A third of hospital patients with CSOM in India had hearing loss better than 40 dB HL, a third had hearing loss 40–60 dB HL and a third had loss > 60 dB HL [7]. In some cases, hearing loss can also be due to erosion of the ossicles, middle ear mucosa thickening or fibrosis or the presence of middle ear effusion [8]. Those with a perforated TM may have additional sensorineural hearing loss, for example, because of presbyacusis or because the presence of CSOM has caused cochlear damage [9] (presumed to be due to inflammatory toxins) [3].

Options for rehabilitation of hearing in those with a tympanic perforation include myringoplasty (with or without ossiculoplasty), conventional air conduction hearing aids, or bone conduction hearing aids. A review of outcomes of myringoplasty in children found that (where measured) 89% achieved functional hearing (thresholds better than 30 dB HL) [10]. However, the majority of the population affected by CSOM lacks access to surgery, due to a complex array of factors, including affordability and availability of surgical expertise [11, 12].

Air conduction hearing aids can rehabilitate hearing loss of any cause and severity up to typically 80 dB thresholds [13], but tend to have low availability in low‐resource settings (largely because of poor availability of audiologists [12] to programme such aids and the affordability of the devices) [14]. Air conduction aids are also relatively contraindicated in the presence of a TM perforation because otorrhoea may prevent their use, or conversely, their use may precipitate or perpetuate otorrhoea [15].

Bone conduction hearing aids are used less often, particularly in low‐income settings, because traditionally such aids have been costly and require surgical implantation. However, recent availability and pilot data have shown the effectiveness of low‐cost non‐implanted bone conduction devices (BCD) such as personal music players or headsets for chronic middle ear effusion in children [16]. This has created an opportunity for this technology to be expanded for use in those with a perforated TM, including in low‐income settings [17]. BCD do not necessarily require programming in those with a purely conductive hearing loss, and so may preclude the need for an audiologist, but are less effective when sensorineural hearing loss is present. Implanted BCDs may be rendered ineffective when bone conduction thresholds are worse than 60 dB HL. For non‐implanted devices, function may be compromised if contact of the device with the skull is suboptimal because of loose fitting or thick overlying soft tissue [18], which can cause up to 20 dB loss of functional gain in high frequencies, and a difference in speech reception threshold of up to 7 dB [19].

To date, there are no data to inform national or international policy or strategy for assistive technology (air or bone conduction hearing aids) for the rehabilitation of hearing loss in people with a perforated TM. Data on the degree and type of hearing loss in the global population with CSOM are limited to individual case series. Here, we undertook a systematic review and meta‐analysis of published literature to evaluate air and bone conduction thresholds in children and adults with a perforated TM.

## 2 Methods

### 2.1 Literature Review of Hearing in Tympanic Perforation: Study Inclusion

The study was registered with PROSPERO (CRD42022255652) [20] and reported in accordance with the PRISMA 2020 standard [21].

We systematically searched published literature on hearing threshold with tympanic perforation on six electronic databases from inception until 1 December 2023: EMBASE, MEDLINE, CINAHL, WHO International Clinical Trial Registry Platform, the Cochrane library and the ISRCTN registry. Medical Subject Headings (MeSH terms) used to interrogate each database were synonyms of ‘hearing loss’, ‘hearing threshold’, ‘chronic suppurative otitis media’ and ‘tympanic membrane perforation’ (Table 1). There were no restrictions on year or language of publication. We included data from non‐English language papers if translated contents could be verified by a professional speaker/reader in that language. We consulted subject matter experts and manually searched reference lists of included publications to identify additional studies that may have been missed by the initial search.

**TABLE 1:** MeSH terms used in bibliographic search.

	1	2	3	4
Concept	Tympanic membrane perforation	Hearing threshold	Hearing loss	Chronic suppurative otitis media, otitis media and cholesteatoma
Actual term searched	‘tympanic membrane perforation’ OR ‘ear drum perforation*’ OR ‘perforation*’	Hearing threshold* OR hearing level* OR audiogram* OR ‘hearing test*’	‘Hearing loss’ OR Deaf* OR ‘Hearing impair*’	‘Chronic suppurative otitis media’ OR ‘Chronic supporative otitis media’ OR ‘CSOM’ OR ‘Chronic otitis media’ OR ‘Otitis media’ OR ‘Otitis media with effusion’ OR ‘glue ear’ OR ‘Cholesteatoma*’

*Note*: Columns 1 and 2 were combined using the operator ‘AND’. Then Columns 1 and 2 were combined with ‘AND’ Column 3 ‘OR’ Column 4 and then finally combined with both Column 3 ‘AND’ 4.

We included studies of adults and/or children (those aged < 18) with TM perforation of whatever cause, from systematic reviews, randomised controlled trials, controlled (non‐randomised) clinical trials or cluster trials, cohort studies, case–control or nested case–control studies, cross‐sectional studies and case series (but excluding case series with fewer than 10 patients). We excluded animal or experimental studies, studies of individuals with otitis media or hearing loss due to named congenital disorders or chemotherapy or radiation‐induced damage and studies of tympanic perforation related to trauma, blast injury or acute otitis media. We excluded studies not reporting audiometric data.

Duplicates studies were identified and removed, and title, abstract and full‐text screening for relevance was independently undertaken by two reviewers (T.H., A.S.) using Endnote (version 20.2.1, Clarivate 2020). Where consensus was not achieved, arbitration was carried out by a third reviewer (M.B.). Studies were assessed for risk of bias using the Murad et al. [22] methodological quality tool and excluded where appropriate.

### 2.2 Review of Hearing in Tympanic Perforation: Data Extraction and Analysis

We recorded data on the characteristics of the study and participants, including age, sex, self‐reported ethnicity, active otorrhea, cause of perforation and disease laterality.

We extracted data on air and bone conduction hearing thresholds, and where this was from interventional studies, we used only pre‐intervention data. We contacted original study authors for clarification or for missing data where needed and possible.

We separated extracted data into that from children and from adults. Where studies included both adult and child participants and data could not be separated, data were excluded (because the differing prevalence of sensorineural hearing loss in adults vs. children could confound results).

We analysed aggregate data on hearing thresholds for mean average, median and inter‐quartile range. Where individual participant data were not available, we used aggregate parameters if these were reported by study authors. We performed a meta‐analysis of extracted data with the ‘meta’ and ‘metaphor’ package for R Studio (RStudio, PBC, Boston, MA) using a random effects model [23], assessing heterogeneity using *I* ^2^ and *𝜏* ^2^ tests, and calculating prediction intervals [24]. The prediction interval can help clinical interpretation of heterogeneity by estimating what true treatment effects or measures might be expected in future settings [25].

A priori subgroup analysis was planned and undertaken for age (paediatric < 18 years and adult ≥ 18 years) and for studies which reported perforation due to CSOM versus other causes, and for income status (World Bank ranking) of the country of study.

## 3 Results

**FIGURE 1:**
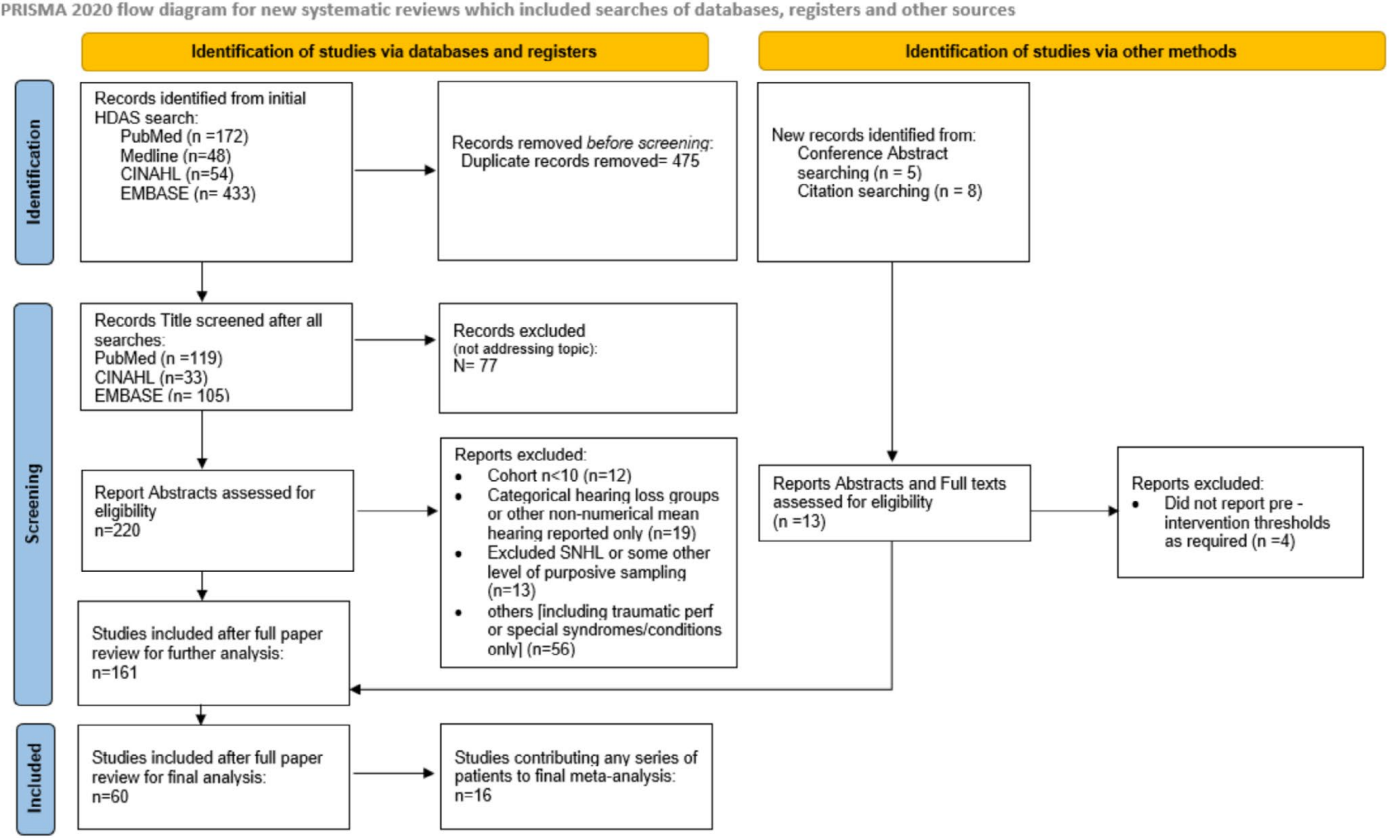
PRISMA diagram of included and excluded studies in review of hearing in tympanic perforation. *Source*: Page et al. [21]. For more information, visit: http://www.prisma‐statement.org/ .

Our search recovered 720 studies, with 235 remaining after de‐duplication, and 141 after screening abstracts and titles (Figure 1). After full‐text review, 60 studies were potentially informative, but on further analysis, only 16 studies included discrete data on hearing thresholds. No studies were excluded due to risk of bias.

In the 16 included studies [26, 27, 28, 29, 30, 31, 32, 33, 34, 35, 36, 37, 38, 39, 40], data for 82 subgroups of patients with hearing thresholds were extracted, comprising 34 adult‐only series, eight child‐only series and 40 mixed‐age series. Differences in how hearing thresholds were recorded (e.g., recorded at each individual frequency or pooled average; measuring bone conduction, air conduction or only air‐bone gap and whether standard deviation was or was not reported) meant that only a few case series were informative for the final meta‐analysis: 16 series measuring adult air conduction thresholds, 13 measuring adult bone conduction, eight child air conduction and four child bone conduction. Data included in the final analysis comprised 1303 bone conduction thresholds and 1700 air conduction thresholds taken from 1394 ears of adults and 380 ears of children.

**FIGURE 2:**
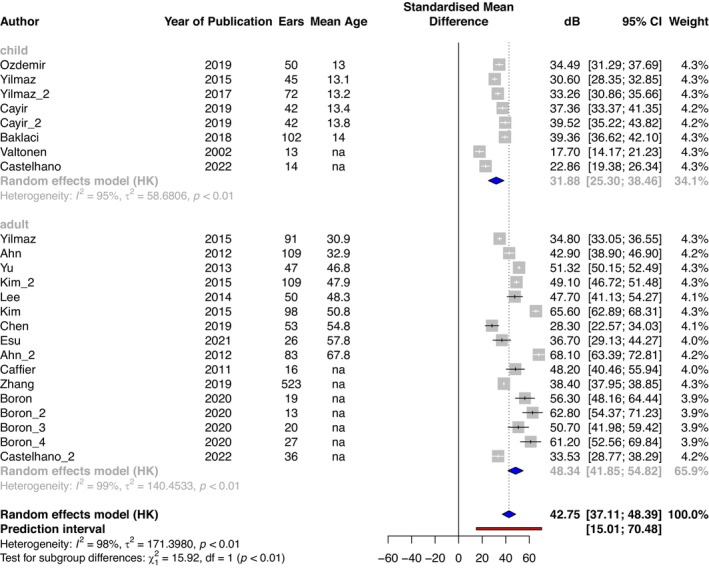
Mean air conduction thresholds (dB) of adults and children with tympanic membrane perforation. Mean of respective series = blue diamond, pooled mean of all included studies = dashed line and diamond, prediction interval = red line. Individual study series are ordered by mean age (years). Where such data were not available (na), the series is at the bottom of the plot.

**FIGURE 3:**
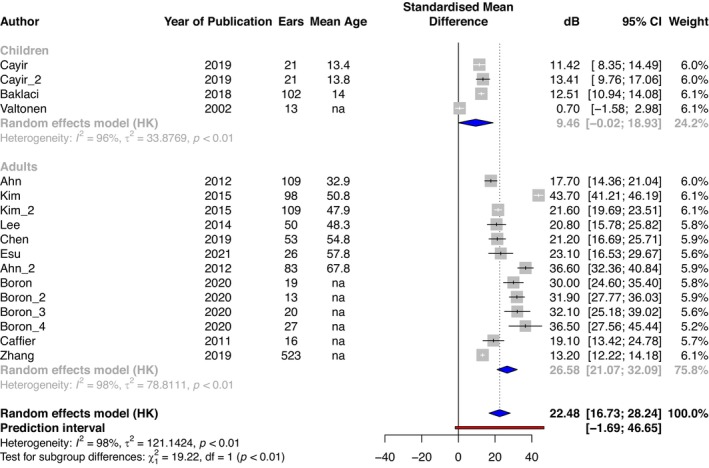
Mean bone conduction thresholds (dB) of adults and children with tympanic membrane perforation. Mean of respective series = blue diamond, pooled mean of all included studies = dashed line and diamond, prediction interval = red line. Individual study series are ordered by mean age (years). Where such data were not available (na), the series is at the bottom of the plot.

Overall mean air conduction threshold where reported was 42.8 dB (Figure 2), with a mean in adults of 48.3 dB (95% CI: 41.9–54.8 dB, *n* = 1320 ears, *I* ^2^ = 99%, *p* < 0.01), and in children of 31.9 dB (95% CI: 25.3–38.5 dB, *n* = 380 ears, *I* ^2^ = 95%, *p* < 0.01). Mean bone conduction threshold where reported was 22.5 dB HL (95% CI: 16.7–28.2 dB) (Figure 3), with a mean in adults of 26.6 dB (95% CI: 21.1–32.1 dB, *n* = 1146 ears, *I* ^2^ = 98%, *p* < 0.01), and in children of 9.5 dB (95% CI: 0.0–18.9 dB, *n* = 157 ears, *I* ^2^ = 96%, *p* < 0.01). The prediction interval (estimates where the true effects would be expected for 95% of similar (equivalent) studies that could be conducted in the future [25]) had bounds of −1.7 to 46.7 dB for bone conduction and 15.0–70.5 dB for air conduction (Figures 2 and 3).

There was an apparent and expected trend of worse hearing with increasing age, but regression demonstrated only weak correlation for both air (*p* = 0.8) and bone conduction (*p* = 0.9). In subgroup analysis, 38 series included perforations associated with CSOM, totalling 2365 ears. There was only one series in children and one in adults evaluating air conduction thresholds in perforations without CSOM, meaning there were insufficient studies for subgroup analysis on this variable. There were also insufficient data to allow analysis on country or regional income status.

## 4 Discussion

We sought to define parameters for air and bone conduction aids as options for the rehabilitation of hearing loss in children and adults with a perforated TM.

For air conduction thresholds, our meta‐analysis included case series for children and adults up to a mean age of 68, and here the upper range of the prediction interval was 70 dB. This implies that 95% of adults up to age 68 years with a perforated TM can potentially benefit from air conduction aids able to amplify to this level. Contemporary air conduction hearing aids can easily provide linear amplification up to a hearing threshold of 60–65 dB [41] and a highest peak gain of 87 dB [42] with average functional gains of 39 dB [43].

For bone conduction thresholds, the meta‐analysis included case series for children and adults also up to a mean age of 68, and here the upper range of the prediction interval was 47 dB. This implies that 95% of adults up to age 68 with a perforated TM can be rehabilitated with a bone conduction aid able to amplify to this level. Implanted bone conduction aids can be effective for those with bone conduction thresholds of up to 60 dB [44]. However, it is noteworthy that in all the case series for children (Figure 3), the maximum upper 95% confidence interval for bone conduction thresholds was 17 dB, and in the case series for adults (where mean age was reported) up to the age of 58, the maximum upper 95% confidence interval limit was 30 dB. This implies that 95% of adults up to age 58 with a perforated TM will be rehabilitated with bone conduction aids able to amplify to this level. External (non‐implanted) bone conduction aids are also likely to be able to amplify to this level, but this is yet to be proven.

These audiological parameters and options for hearing rehabilitation are summarised in Table 2.

**TABLE 2:** Suggested options for assistive technology for the rehabilitation of individuals with tympanic perforation according to audiological parameters.

Audiological parameter	Assistive technology option	Predicted candidates
Air conduction thresholds up to 80 dB	Air conduction aid	Majority of adults and children
Bone conduction thresholds up to 60 dB	Implanted bone conduction aid	Majority of adults and children
Bone conduction thresholds up to 30 dB	Non‐implanted bone conduction aid	Majority of adults and children up to (at least) age 58

Heterogeneity (*I* ^2^) was as high as 99% for adult bone conduction thresholds. We explored this by calculating 95% prediction intervals, which were calculated with bounds of −1.7 to 46.7 dB for bone conduction and 15.0–70.5 dB for air conduction (see Figures 2 and 3). If there were an absence of any between‐study heterogeneity, this prediction interval would coincide with the respective confidence intervals [25]. However, here, even though our prediction interval covers a wider range, importantly, even with these boundaries, our analysis implies that most adults and children with a perforated TM are likely to benefit from a BCD.

We are cognisant that the majority of the global population with perforated TMs live in low‐ and middle‐income countries. Access to assistive technology for such populations is limited, for example, in some areas of Africa, air conduction aids have been estimated to reach only 3% of those who may benefit [45], although recent legislative changes in the United States to enable over‐the‐counter sales of such technology [46] may improve availability.

The option for non‐implanted bone conduction aids is particularly relevant for low‐resource settings, where such options could be inexpensive and not require the presence of a medical professional. Additional research should seek to clarify differences in head and skull morphology and soft tissue depth measurements to inform the future design of such devices for different age groups. The impact on sound conduction of external BCD from soft tissue thickness and pressure at contact points is also an area for future research.

At present, extrinsic BCD are largely offered as a pre‐implantation trial for those patients considering implanted bone‐anchored hearing devices [47], and their sound transmission is not as good as implanted devices [48], with studies showing up to 20%–40% lower speech recognition [19]. Any proposed external device would need real‐world evaluation of candidacy and performance.

There are limitations to our analysis. Although our search yielded community and observational studies, all studies included in the meta‐analysis were exclusively hospital populations, and it is possible disease severity in this group is greater than in the community. Factors such as lower socio‐economic status and the presence of otorrhoea may also associate with disease and hearing severity [49], but in our dataset were poorly recorded, precluding analysis on such variables. We have not reported frequency‐specific mean and median hearing thresholds because of the poor granularity of data in included studies. Related to this, it is possible that bone conduction levels may be artificially elevated (particularly at 2 kHz frequency) due to the Carhart effect [50], although this would be unlikely to affect our conclusions by an appreciable amount.

## 5 Conclusion

Our analysis has demonstrated the prediction interval for expected hearing thresholds for adults and children with a perforated TM. The findings suggest that preserved cochlear function is likely in children, and the mean hearing threshold in both adults and children suggests the majority of people in both groups will benefit from air or bone conduction assistive technology. The parameters and options we have outlined should help in the implementation of assistive technology to rehabilitate hearing loss and improve quality of life in the millions of children and adults across the world affected by a perforated TM.

## Author Contributions

T.H. and M.B. conceptualised the article. T.H. developed the search strategy. T.H. and A.S. appraised papers. T.H. analysed the data and wrote the original draft of the article. T.H., K.M. and M.B. acquired funding. T.H., A.S., K.M. and M.B. provided critical appraisal and editorial contributions. All authors approved the final submitted version. T.H. agreed to be accountable for all aspects of the work.

## Ethics Statement

The authors have nothing to report.

## Conflicts of Interest

The authors declare no conflicts of interest.

## Data Availability

The data that support the findings of this study are available by contacting the corresponding author.

## Appendix 1

### Additional Methods Outcome Measures

Additional outcomes measures will be

- Correlation of hearing threshold with predetermined variables and exposures if details provided:
  1. Sex.
  2. Age (children (< 18 years) vs. adult).
  3. Known previous ototoxic medication exposure.
  4. Active otorrhea.
  5. Comorbidities.
  6. Community versus hospital or health‐system based population.
- Whether the hearing thresholds are different for patients with perforations that are:
  1. Unilateral versus bilateral.
  2. Due to tympanic membrane perforation versus cholesteatoma/CSOM versus otitis media with effusion (glue ear).
  3. Measured with pure tone audiograms or play audiometry (when age matched).
- Whether the hearing thresholds are different for studies from different settings.
  1. World Bank country classifications by income level: 2021–2022 (low‐income, lower middle‐income, higher middle‐income and high‐income countries) (based on status at time of study rather than current).
  2. WHO regions (African, Americas, South‐East Asian, European, Eastern Mediterranean and Western Pacific Regions)

### Additional Methods Criteria for Exclusion of Studies

Studies of self‐reported, subjective hearing loss or where data specifically described hearing assessments using alternative audiological assessments, that is, tuning forks, automated auditory brainstem response (ABR) or automated otoacoustic emissions (or other screening rather than diagnostic bone conduction methodology) were all excluded.

We excluded studies that only reported categorical threshold severities and also excluded any study where the inclusion criteria necessitated exclusion a priori if participants had any degree of conductive or sensorineural hearing loss, as this would artificially exaggerate the anticipated mean thresholds.

### Complete Data Extraction Items (*n* = 66)

Study no.

Title

Email

Author

Journal

Year

Publication type

Intervention

Sample

Ears

Adults

Mean age

Age SD

Mean air conduction threshold

AirSD

AirSEM

Median air IQR

Mean bone conduction threshold

BoneSD

Median bone

Median bone IQR

Air range

Bonerange

Air‐bone gap

AirbonegapSD

AirbonegapSEM

Air and bone conduction means (SD) or median (IQR) for each frequency:

- 250 Hz
- 0.5 kHz
- 1 kHz
- 2 kHz
- 3 kHz
- 4 kHz
- 6 kHz
- 8 kHz

PTA used?

Sex reported?

Otorrhea reported?

Comorbidities

Hospital or community

Unilateral or bilateral

CSOM

Country of study

World Bank region

Worldbank status

Ethnicity reported

Ethics documented?

### Risk of Bias

See Tables A1 and A2.

**TABLE A1:** Risk of bias summary of included studies in the final meta‐analysis based on Murad et al.

Study	Year	Age of series	Selection	Ascertainment	Causality	Reporting
Valtonen [26]	2002	Child	*	*	*	*
Caffier [27]	2011	Adult	*	*	*	*
Ahn [28]	2012	Adult	*	*	*	*
Yu [29]	2013	Adult		*		*
Lee [30]	2014	Adult		*		*
Kim [51]	2015	Adult	*	*		*
Yilmaz [31]	2015	Child and adult	*	*	*	*
Yilmaz_2 [32]	2017	Child		*	*	*
Baklaci [33]	2018	Child		*	*	*
Çayir [52]	2019	Child		*	*	*
Chen [34]	2019	Adult	*	*	*	*
Özdemir [40]	2019	Child	*	*	*	*
Zhang [35]	2019	Adult		*	*	*
Boroń [36]	2020	Adult		*	*	*
Esu [37]	2021	Adult		*	*	
Castelhano [38]	2022	Child and adult		*	*	*

*Note*: Methodological quality and synthesis of case series and case reports tool. * indicates satisfactory quality [22].

**TABLE A2:** Final 16 studies included in meta‐analysis.

Study no.	Study first author	Authors	Title	Journal	Year
4	Valtonen	H. J. Valtonen	Otological and Audiological Outcomes Five Years After Tympanostomy in Early Childhood	*Laryngoscope*	2002
16	Caffier (41)	Caffier, B. Sedlmaier, H. Haupt, O. Göktas, H. Scherer and B. Mazurek	Impact of Laser Eustachian Tuboplasty on Middle Ear Ventilation, Hearing, and Tinnitus in Chronic Tube Dysfunction	*Ear & Hearing*	2011
18	Ahn (42)	Ahn Yun Suk, B. Ji Sun and K. Do Yoon	Postoperative Results of Tympanoplasty With Mastoidectomy in Elderly Patients With Chronic Otitis Media	*Annals of Otology, Rhinology & Laryngology*	2012
23	Yu (43)	F. Yu	A Novel Technique for Reconstruction of the Posterior Wall of the External Auditory Canal and Tympanum Using Pedicled Temporalis Myofascia	*Acta Oto‐Laryngologica*	2013
24	Lee (44)	Lee, S	Vestibular Evoked Myogenic Potential According to Middle Ear Condition in Chronic Otitis Media With Tympanic Membrane Perforation	*Acta Oto‐Laryngologica*	2014
29	Kim	S.H. Kim	Pre‐ and Postoperative Clinical Findings of Tympanomastoid Surgery in Female Divers (Haenyeo) of Jeju Island With Chronic Otitis Media	*Acta Oto‐Laryngologica*	2015
30	Yilmaz	M. S. Yilmaz	Comparison of the Anatomic and Hearing Outcomes of Cartilage Type 1 Tympanoplasty in Paediatric and Adult Patients	*European Archives of Oto‐Rhino‐Laryngology*	2015
37	Yilmaz_2	Yilmaz M. S. et al., A. Kara, M. Guven, D. Demir and U. Erkorkmaz	Assessment of the Factors That Affect the Anatomic and Functional Success of Cartilage Tympanoplasty in Children	*The Journal of Craniofacial Surgery*	2017
38	Baklaci	Baklaci, I. Guler, I. Kuzucu, R. O. Kum and M. Ozcan	Type 1 Tympanoplasty in Paediatric Patients: A Review of 102 Cases	*BMC Paediatrics*	2018
45	Cayir	Cayir S.	Type 1 Tympanoplasty in Paediatric Patients: Comparison of Fascia and Perichondrium Grafts	*International Journal of Paediatric Otorhinolaryngology*	2019
46	Chen	S.L. Chen	Factors Affecting the Treatment Outcomes of Myringoplasty in Patients With Small Tympanic Membrane Perforations	*European Archives of Oto‐Rhino‐Laryngology*	2019
47	Ozdemir	D. Özdemir	Transcanal Endoscopic Type 1 Cartilage Tympanoplasty in Children	*Turkish Archives of Otorhinolaryngology*	2019
44	Zhang (52)	J. Zhang	A Multi‐Centre Clinical Retrospective Study on the Therapeutic Effect of Endoscopic Myringoplasty	*Chinese Journal of Otorhinolaryngology Head and Neck Surgery*	2019
48	Boron	A. Boroń	Long‐Term Results of a Hearing Test in Patients Operated for Chronic Otitis Media	*The Polish Otolaryngology*	2020
54	Esu	Esu, S. Tamii, M. Masuda, Y. Iino and N. Yoshida	Effectiveness of Myringoplasty in Patients With Eosinophilic Otitis Media	*Auris Nasus Larynx*	2021
58	Castelhano	Castelhano L, Correia F, Colaco T, L. Reis and P. Escada	Tympanic Membrane Perforations: The Importance of Aetiology, Size and Location	*European Archives of Oto‐Rhino‐Laryngology*	2022

### Additional Results Data About Reviewed Studies

- Fifty‐seven articles were written in English and three were in Mandarin (Chinese).
- The oldest study was from 1982, but 49 papers were from 2010 onwards.
- Only eight studies were observational, the other studies assessed pre‐op thresholds from intervention studies.
- Because of the number of studies that were based on surgical pre‐ and post‐op results, we expected most studies to assess the hearing of inpatient surgical candidates, but only 45 studies clarified an inpatient or hospital‐based population. Only Jensen et al. [53] was conducted in a community setting (in Greenland).
- Only nine studies looked at data from children to adults discretely.
- Only six studies reported thresholds discretely by sex.
- No studies reported participant race or ethnicity.
- In total, 20 papers had no record of research ethical or institutional review. Beyond issues of research integrity, we feel that issues with the heterogeneity of study data or lack of transferability of data between series could be improved if more institutional review boards were consulted for study design, advice and guidance before research started.
- Overall, the studies in the wider review were authored from 24 countries. The greatest number of contributing studies to the review came from India (*n* = 12), with at least three studies from Korea, Japan, Iran, Bangladesh, the United States, China and Turkey, respectively.
- World Bank country classifications by income level: (low‐income, lower middle‐income, higher middle‐income and high‐income countries) (based on status at time of study rather than current) revealed that all the meta‐analysed series were extracted from studies conducted in high or upper middle‐income settings (plus Taiwan which is unclassified).
- Even in the wider review, there were only 25 within‐study series from lower middle‐income settings and no studies whatsoever from low‐income settings. Each of the WHO regions was represented (African, Americas, South‐East Asian, European, Eastern Mediterranean and Western Pacific Regions), but there was only one study included from the African region.
